# *KIR* Gene Content in Amerindians Indicates Influence of Demographic Factors

**DOI:** 10.1371/journal.pone.0056755

**Published:** 2013-02-25

**Authors:** Danillo Gardenal Augusto, Bruno Zagonel Piovezan, Luiza Tamie Tsuneto, Sidia Maria Callegari-Jacques, Maria Luiza Petzl-Erler

**Affiliations:** 1 Laboratório de Genética Molecular Humana, Universidade Federal do Paraná, Curitiba, Brazil; 2 Laboratório de Imunogenética, Universidade Estadual de Maringá, Maringá, Brazil; 3 Departamento de Estatística, Universidade Federal do Rio Grande do Sul, Porto Alegre, Brazil; University of Hawaii Manoa, United States of America

## Abstract

Although the *KIR* gene content polymorphism has been studied worldwide, only a few isolated or Amerindian populations have been analyzed. This extremely diverse gene family codifies receptors that are expressed mainly in NK cells and bind HLA class I molecules. KIR-HLA combinations have been associated to several diseases and population studies are important to comprehend their evolution and their role in immunity. Here we analyzed, by PCR-SSP (specific sequencing priming), 327 individuals from four isolated groups of two of the most important Brazilian Amerindian populations: Kaingang and Guarani. The pattern of *KIR* diversity among these and other ten Amerindian populations disclosed a wide range of variation for both *KIR* haplotypes and gene frequencies, indicating that demographic factors, such as bottleneck and founder effects, were the most important evolutionary factors in shaping the *KIR* polymorphism in these populations.

## Introduction

Killer cell immunoglobulin-like receptors (KIR) are expressed on natural killer (NK) cells and subsets of T cells playing an important role in innate immunity and influencing also the course of adaptive immune responses. These cells are regulated by many activating and inhibitory signals, including signals delivered by the interaction between KIR and their ligands [1]. KIR are encoded by genes in the 19q13.4 genomic region [2] and classified according to the number of extracellular Ig-like domains as KIR2D or KIR3D and according to the cytoplasmic tail as L (long) or S (short) [3]. These structural features correlate with broad functional differences and with the characteristics of cognate ligands. Most KIR with long cytoplasmic tails transduce inhibitory signals and those with a short cytoplasmic tail are activating. The exception is KIR2DL4 that has the ability to transduce both activating and inhibitory signals [4].

The known ligand of KIR2DL1 is HLA-C2 while KIR2DL2 and KIR2DL3 interact with HLA-C1 and some C2 allotypes [5]. KIR2DS1 weakly interacts with HLA-C2 [6], [7]. KIR3DL1 recognizes HLA-B and HLA-A molecules with the Bw4 epitope [8] and it has been suggested that KIR3DS1 also binds the Bw4 epitope [9], [10]. Although HLA-B epitopes are currently considered the most important KIR3DL1/S1 ligands, we recently suggested that both HLA-A and B allotypes may be equally important for NK function [11], which corroborates the suggestion about HLA-A and HLA-B having compensatory function in the engagement of KIR receptors and being a KIR-driven functional genetic block [12].

The distinction between the *KIR* genes and alleles is not always clear. Seemingly *3DL1* and *3DS1* (for simplicity, we will from now on omit KIR from the symbols of the genes and their products) are alleles of the same locus [13], as *2DL2* and *2DL3* are [14]–[16]; however, some haplotypes contain both *3DL1* and *3DS1* or lack both [14], [17]. Further, several haplotypes contain two *2DL5* genes, named *2DL5A* and *2DL5B* and these same haplotypes may contain one or two *2DS3* genes [18], [19]. Also, the distinction between *KIR* genes and pseudogenes became blurred since it has been recognized that the *3DP1* pseudogene has one functional allele [20] and the *2DL4* and *2DS4* genes have common non-functional alleles [21], [22]. These uncertainties are side effects of the rapid evolution of the *KIR* genomic region at which the evolutionary recent expansions and contractions by unequal crossover played an important role [23].

Numerous haplotypes differing for gene content have been described in human populations; they are classified in haplogroups A and B [16]. Group A haplotypes are usually characterized by the following genes and pseudogenes, listed in centromeric to telomeric orientation: *3DL3*, *2DL3*, *2DP1*, *2DL1*, *3DP1*, *2DL4*, *3DL1*, *2DS4*, *3DL2*. However, a few A haplotypes missing one or more of these genes above have been described (allelefrequencies.net [24]). The group B haplotypes differs for gene content especially by the presence of more genes encoding short-tailed KIR. The genes *3DL3*, *2DL4* and *3DL2* and the *3DP1* pseudogene are almost always present in both the groups A and B haplotypes in the same relative orientation and therefore occupy the so-called framework loci [25], [26]. One or the other of the “allelic genes” *2DL2/2DL3* and *3DL1/*3DS1 are also present in virtually all *KIR* haplotypes.

The evolutionary peculiarities of the *KIR* gene family, the high degree of genetic polymorphism and the crucial function of KIR in immunity stimulated the search for implications of their variability. The frequencies of individual *KIR* genes and haplotypes differ among populations and the causes of this variation are still a matter of debate. Particularly, the relative influence of different forms of natural selection and of demographic contingencies remains to be established. The motivation for a deeper understanding of KIR diversity and function is high since it has been recognized that *KIR* and *HLA* genotypes influence a person’s susceptibility to infectious and autoimmune diseases and the outcome of transplantation in specific clinical settings. In order to achieve insight on some of these questions, analyses of populations all over the world are of prime importance. The aims of this work were to describe the diversity of *KIR* genes and haplotypes for gene content variation in four endogamic Amerindian populations and to search for the causes of the observed variation by comparisons between these and with other previously described Amerindian populations.

## Results

### Gene Frequencies

Comparing gene frequencies among populations, especially genetically isolated groups, is a powerful tool to understand the possible causes of variations in a certain gene or gene family and it may help to trace the history of the populations. The frequencies we see in extant populations were shaped by both natural selection and demographic factors, such as migrations, bottlenecks and other factors, along human evolution. To evaluate the relative importance of natural selection and demographic factors for *KIR* gene content polymorphism in Amerindian populations, we described the *KIR* polymorphism in sizeable samples of two of the most important Amerindian groups and compared their diversity with that of other isolated and urban populations.

**Figure 1:**
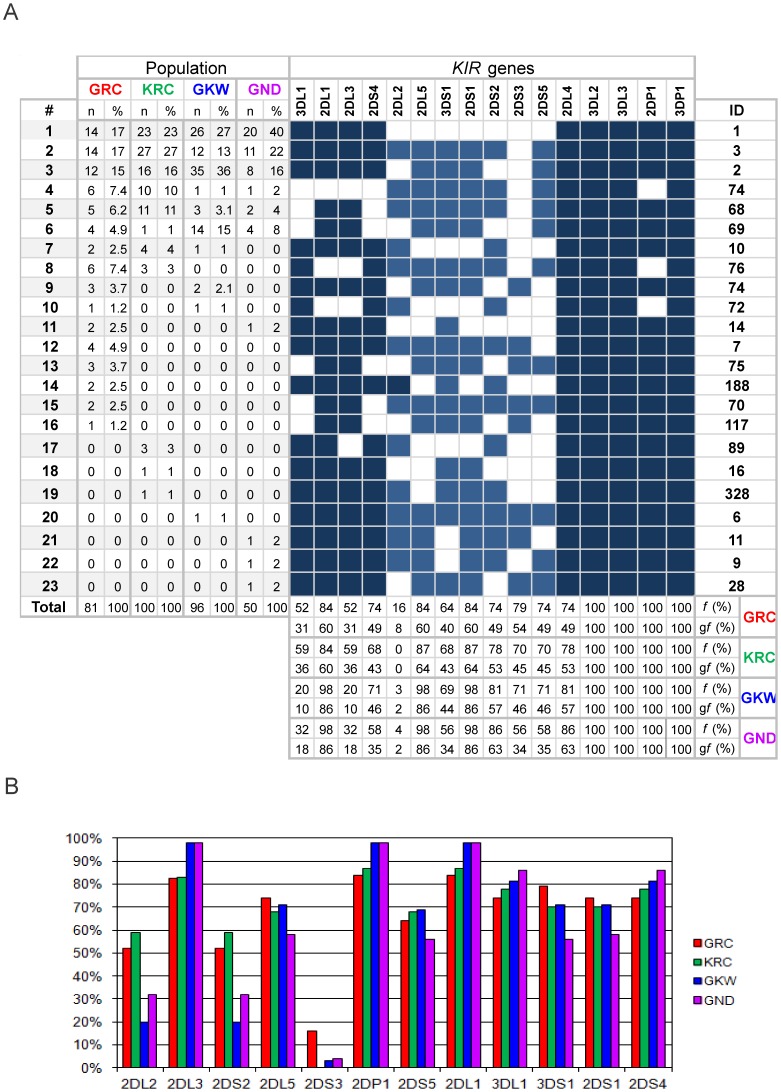
Frequency of *KIR* profiles and of individual *KIR* genes in the Kaingang and Guarani populations. **1A** – The frequencies of KIR profiles are on the left side and the frequencies of individual genes are on the bottom. Filled boxes indicate presence of the gene and blank boxes, absence. In dark blue are genes typically from haplotypes A and light blue, from haplotypes B. n = number of individuals; *f* = carrier frequencies; *gf* = gene frequency; ID = identification number according allelefrequencies.net [24]. **1B** - Phenotypic frequencies of each KIR gene and pseudogene in the Kaingang and the three Guarani populations. Genes observed in all individuals are not shown.

The framework genes *3DL2*, *3DL3* and *2DL4* and pseudogene *3DP1* occurred in all individuals. The other genes and the *2DP1* pseudogene were observed in all four populations at varying frequencies (Figure 1a and 1b), with the only exception of *2DS3* that was absent in the Kaingang (KRC). In other Amerindians, the frequency of *2DS3* varies from zero in Wichi, Yucpa, Bari and Tarahuama to 20% in Warao [27]–[29]. Almost all populations worldwide have the *2DS3* gene at considerable frequencies (>20%), the highest frequency being reported for Australian Aborigines (81%) [24]. Southern and Central Asian populations, as some Indian and Pakistani groups, also present high frequency of *2DS3,* while most Eastern Asian including Japanese, Korean and Chinese populations present frequencies lower than 20% [24].

The two-domain *2DS4* is the only short tailed activating gene present in the haplotype A, the most frequent haplotype worldwide [16]. This gene has a frequent group of alleles characterized by a deletion of 22bp in exon 5 (also named *KIR1D* [26], [30]). The alleles thus far described, that are included in that group are 2DS4*003, *004, *006, *007 and the uncommon *008, *009, *010, *012 and *013 alleles. Alleles that do not present the 22pb deletion are the common 2DS4*001 alleles and the rare *011, *014, *015 alleles (IPD database and allelefrequencies.net; [24], [31]). Describing this group is important not only in the functional context, because individuals having two A haplotypes with *KIR1D* alleles may completely lack functional activating genes, but also because it is an additional tool for inferring haplotypes. In addition, distinguishing the two groups of *2DS4* alleles provides information for more refined comparisons between populations. In the study populations, the frequency of this deletion varied from zero in the Guarani-M’byá (GRC) to 24% in the Guarani-Ñandeva (GND; Table 1), and similarly to the *2DS3* gene frequencies, *2DS4* allele groups showed a wide range of variation among Amerindians.

**Table 1:** Frequencies of the two *KIR2DS4* allelic groups characterized by the 22bp indel and absence of the *KIR2DS4* gene.

Alleles and genotypes	KRC (n = 100)	GRC (n = 81)	GKW (n = 96)	GND (n = 50)
***Allele groups***				
2DS4*001 a *(ins)*	0.41	0.49	0.49	0.39
2DS4*003 -*007 b *(del)*	0.12	0	0.08	0.24
absence of *2DS4 (abs)*	0.47	0.51	0.43	0.37
***Genotypes***				
*ins/ins+ins/abs*	0.55	0.74	0.66	0.44
*ins/del*	0.07	0	0.06	0.22
*del/del+del/abs*	0.16	0	0.09	0.2
*abs/abs*	0.22	0.26	0.19	0.14

n: number or individuals in the population sample; ins: insertion; del: deletion; abs: absence of the gene;

a Alleles of the 2DS4*001 group: the common *00101 allele and the rare alleles *001xx (other than *00101), *011, *014, *015;

b Alleles of the 2DS4*003 -*007 group: *003, *006, *007, *004 and the rare *008, *009, *010, *012, *013 alleles.

**Figure 2:**
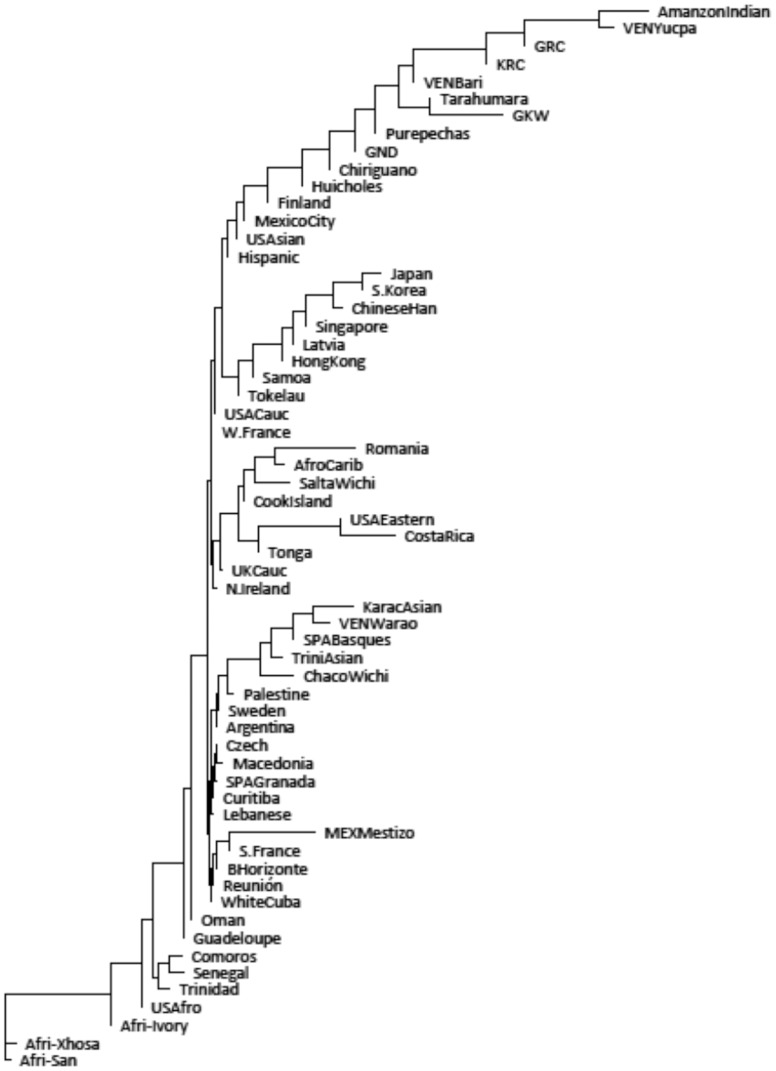
Neighbor joining dendrogram of Nei’s genetic distances among populations, based on the KIR gene frequencies. Gene frequencies available on allelefrequencies.net [24].

**Figure 3:**
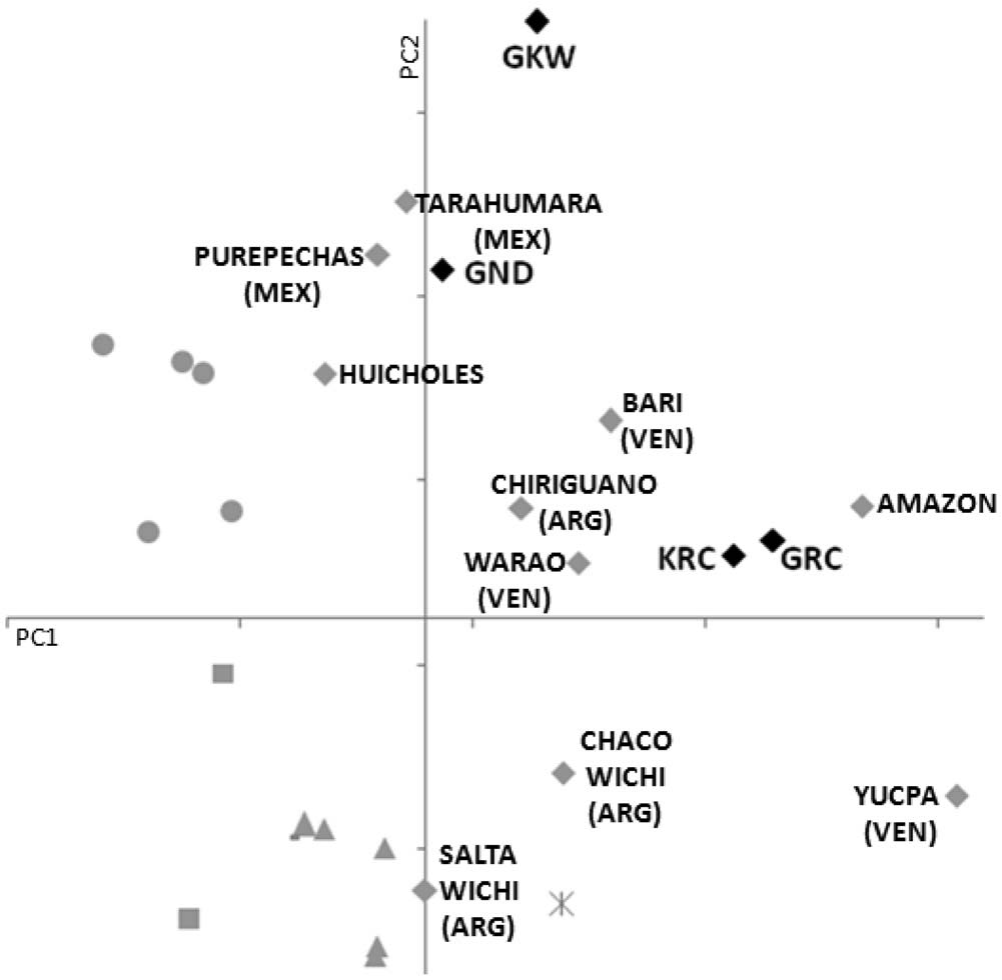
Principal component analysis of worldwide populations including the Kaingang and the three Guarani populations. Triangle = Europeans and Euro-descendants; square = Africans and African-descendants; circle = Eastern Asians; diamond = Amerindians; asterisk = Asian Indians. Gene frequencies are available on allelefrequencies.net [24]. The cumulative percentage of variance represented by the first two principal components is 64%.

We used KIR gene frequencies to measure differences between the populations and to compare them to other previously described groups. We used two different approaches: (1) estimating genetic distances and drawing a dendrogram and (2) performing a principal component analysis. The first method is a quantitative approach that estimates genetic distances between populations based on their gene frequencies and show these distances in a dendrogram that groups populations presenting similar frequencies. The second method converts a set of observations of possibly correlated variables (here, the frequencies of the different *KIR* genes) into a set of values of linearly uncorrelated variables called principal components. The first principal component has the largest possible variance, accounting for as much of the variability in the data as possible, and each succeeding component has the largest variance under the constraint that it be uncorrelated with the preceding components. Similar populations will be plotted nearby in a XY graph. Both approaches are powerful to compare populations and can corroborate the results of each other. All Amerindian populations grouped together in the dendrogram (Figure 2), except the Wichi and Warao. The Mexican and other Hispanic populations, which have known Amerindian ancestry, also grouped in the same clade. The exception in this clade is the Finns, which were not expected to group with Amerindians. The two Whichi populations appear at the periphery of the Amerindian cluster in the first two principal components plot (Figure 3), not far from the core of the group. The first two principal components depicted represent 64% of the diversity among populations.

Next, we analyzed the frequencies of KIR receptors combined with the frequencies of their known HLA ligands in every population. As *KIR* and *HLA* genes are not linked, we would expect that all possible *KIR/HLA* combinations occur according to the product of their frequencies, unless some force, like natural selection, is increasing or decreasing certain combinations. The joint frequencies of *3DL1* and of *3DS1* with the Bw4 motif of HLA-A and B molecules; of *2DL1* and *2DS1* with the *C2* group of *HLA-C* alleles, and of *2DL2*, *2DL3* and *2DS2* with the *C1* group of *HLA-C* alleles were compared to the frequencies expected at equilibrium, under the hypothesis of selective neutrality. For all combinations the observed and expected frequencies did not differ significantly (P>0.05), so no evidence of natural selection on the KIR-HLA combinations was detected by this approach. However, the number of individuals having none of the known functional KIR-HLA combinations was zero in the population samples analyzed, an indication that natural selection could be maintaining at least one KIR-HLA functional pair per individual.

### Profiles and Haplotypes

*KIR* profiles, the set of *KIR* genes present in one individual, also named phenotypes or genotypes throughout the literature, can be used as markers of certain ancestral groups, being more informative than frequencies of individual genes. We found 10 to 16 different profiles in the study populations (Figure 1a). Only 4 to 6 of these profiles occurred at frequencies higher than 5% and the three most common accounted for 49% to 78% of the variation in the Amerindian populations studied. The four populations shared only 6 of the 23 profiles observed. Of the remaining profiles, 12 were seen in just one individual.

The three most common profiles of the Guarani and Kaingang are common in many other populations. The A/A profile (ID 1 in allelefrequencies.net [24]) presented the highest frequency in all four populations of this study (17% to 40%) and it was observed in populations of all continents, mostly at high frequencies. The most noticeable exception is the Australian Aborigines (1.5%) [32], however, in some Asian Indian populations this profile is also uncommon (2.9% to 5.6%) [33], [34]. The frequency of haplogroup A varies considerably among Amerindians. The high frequencies observed in the Guarani and the Kaingang are in marked contrast to the very low frequency reported for Amazonian Amerindians (2.5%) [35] but not for Amerindians from Argentina (ca. 31%) [28].

**Figure 4:**
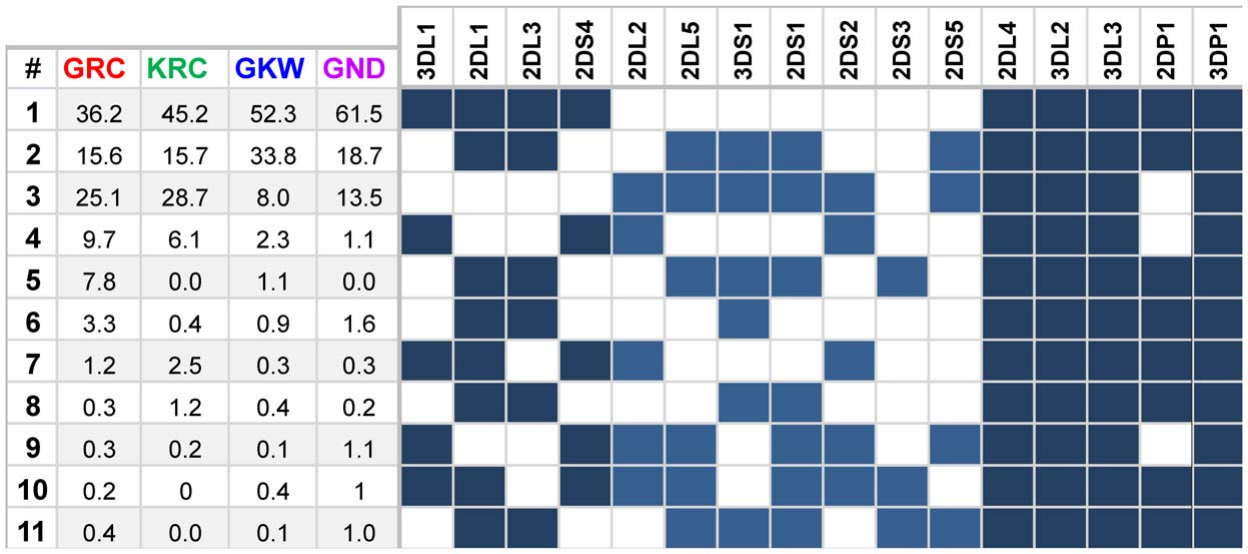
Haplotypes and their frequencies in the four Amerindian populations analyzed. Filled boxes indicate presence of the gene and blank boxes, absence. In dark blue are genes typically from haplotypes A and light blue, from haplotypes B.

The worldwide highest frequencies thus far reported for the A/B #3 (ID 2) profile (up to 41.5%) were seen in Amerindian populations from Mexico and Venezuela [27], [29]. This profile is the most common in the Guarani Kaiowá (GKW) whose frequency is the second highest thus far seen worldwide (36.5%). The frequencies in the other three populations of the present study (15%–16%) are similar to those of several other Amerindian groups, excepting the Wichi in Argentina (5%). This profile occurs in all continents and in most populations the frequency is situated in the 5%–15% interval. We interpret this profile as a result from the heterozygous combination of haplotypes Hap1 and Hap2 (Figure 4).

The A/B #2 (ID 3) profile was the most frequent in the Kaingang group (27%) and is amongst the three most common profiles also in the other study populations (13% to 22%). It is characterized by the absence of *2DS3* gene and its frequency varies from 12.5% to 37.7% in Amerindian populations, which present the highest frequencies of this profile worldwide. It is seemingly absent in Africa and presents low to intermediate frequencies (typically between 2% and 5%) in other continents. Considering the haplotypes inferred, this profile is compatible with genotype Hap1/Hap3 or Hap2/Hap4. Taking into account the haplotypic frequencies and the frequency of the profile, probably most of the ascertained individuals have genotype Hap1/Hap3. The expected proportion of these two genotypes in the total population sample is 0.88 versus 0.12, respectively.

The profiles #4 and #5 (ID 74 and 68, respectively) presented frequencies of 10% and 11% in the Kaingang population, occurring at lower frequencies in the three Guarani groups. Both are shared with other Amerindian populations, but only the Warao in Venezuela present a similarly high frequency (10%) of profile #5 and only in the Yucpa profile #4 is commoner than in the Kaingang (27.7%, the highest thus far reported). In non-Amerindians, profile #4 is virtually absent, while profile #5 was observed at low frequencies (1%–2%) in some populations of other continents excepting Africa (allelefrequencies.net [24]).

Haplotypes are more informative markers than single genes or even profiles for tracing the origins and dispersion of populations through migrations. Moreover, analysis of haplotypes in populations worldwide would help to understand the evolution of the *KIR* gene diversity. Inference of *KIR* haplotypes from heterozygote *KIR* genotypes is hampered in absence of informative family data, because the absence of single genes is a recessive condition. Consequently, distinction between heterozygosity and homozygosity is not possible when a given gene is present; only in the case of its absence homozygosity is certain. Even though, in the present study haplotype inference was facilitated because diversity seen in the Amerindian populations is lower than in the large exogamic populations usually analyzed, thus the reliability of the EM algorithm to identify the haplotypes increases. A total of 11 haplotypes was inferred when the total sample was analyzed using the criteria described in Material and Methods and software Haplo-IHP [36]. The number of haplotypes varied from 8 in the Kaingang to 10–11 in the three Guarani groups (Figure 4). The four most frequent haplotypes were shared by the study populations and accounted for 87% (in GRC) to 95%–96% (in GKW, GND and KRC) of the haplotypes inferred.

Bernstein’s method can be used to estimate gene or allele frequencies when it is not possible to distinguish between heterozygosity and homozygozity due to presence of at least one recessive allele. It assumes that the genotypes occur at frequencies expected in Hardy Weinberg equilibrium. We used this method (m1) to estimate the frequencies of haplogroups A and B, because it was not possible to differentiate between the A/B and B/B genotypes. Starting with the observed frequency of A/A homozygotes, we estimated that the haplogroup B frequencies in Guarani varied from 37% in the Guarani-Ñandeva to 58% in Guarani M’byá. In the Kaingang, the haplogroup B frequency was 52% (Table 2). We also estimated the haplogoup frequencies by counting the haplotypes after haplotype inference (m2). The frequencies obtained by these two estimates did not differ significantly (P = 0.75 for Kaingang; P = 0.65 for Guarani M’byá; P = 1.00 Guarani Kaiowá for and P = 0.87 for Guarani- (Table 2). The similarity of the haplogroup A and B frequencies obtained by the two methods provides indirect evidence for the reliability of haplotype inference in this study and of Hardy-Weinberg equilibrium for *KIR* genotypes.

**Table 2:** Frequencies of haplogroups A and B estimated using two methods, in the Kaingang and Guarani populations.

	Method	Haplotype A	Haplotype B
GRC (n = 81)	m1	0.42	0.58
	m2	0.36	0.64
KRC (n = 100)	m1	0.48	0.52
	m2	0.45	0.55
GKW (n = 96)	m1	0.52	0.48
	m2	0.52	0.48
GND (n = 50)	m1	0.63	0.37
	m2	0.62	0.38

n = number of individuals; m1 = method 1– Bernstein’s formula, which assumes Hardy-Weinberg equilibrium; m2 = method 2– direct counting of the inferred A and B haplotypes.

## Discussion

Our results indicate that demographic factors had a major influence in shaping the gene content variation in these indigenous populations. Several observations lead to this conclusion. First, the frequencies of *2DS3* differ between the study populations and generally among the Amerindians in South America. The frequency of *2DS3* differs significantly (P<0.001) between the Kaingang and the Guarani, varying from zero in the Kaingang to 8% in the Guarani-M’byá. Furthermore, the frequency of the group of alleles which share the 22 bp deletion in *2DS4* differs among the study populations (Table 1; P<0.001) varying from zero (Guarani M’byá) to 24% (Guarani Ñandeva). The frequencies differed even among the Guarani populations, whom share the same close ancestry but live in isolated areas. These observations are compatible with founder and bottleneck effects along the history of these populations.

It is suspected that the variation of the presence/absence of *KIR* genes (especially the activating ones) and frequencies of alleles may have arisen because of natural selection in response to pathogens [34]. Yet, our results did not corroborate the hypothesis that, because of harsher environmental challenges faced by far migrating groups such as the ancestors of American, Australian and Indian natives, the frequency of activating *KIR* and so of haplogroup B would be higher [34]. This proposal was based on the higher frequency of haplogroup B observed in several populations of these geographic regions. What we see in Amerindians instead is a wide range for haplogroup B frequency, which varied from 36% in Huicholes to 84% in Amazonian Indians [27], [35], and from 37% to 58% in the four populations studied here. We consider that the pattern seen in Amerindians is more compatible with a major influence of founder effects and genetic drift than with a scenario of positive or frequency dependent selection increasing the frequency of haplogroup B.

The impact of demographic factors is further corroborated by the frequencies of profiles and inferred haplotypes. Profiles #2 and #3 vary from 13% to 22% and 15% to 36% respectively in the Guarani groups (Figure 1a) and haplotypes #2 and #3 vary from 16% to 34% and 8% to 25%, respectively, in the same populations (Figure 4). In addition, the presence of only few profiles and haplotypes that represent most of the diversity of each population is compatible with predominant roles of founder and bottleneck effects in reducing genetic diversity, and with absence or low gene flow probably due to the cultural and reproductive isolation of these populations.

The frequencies of the genes, haplotypes, haplogroups and profiles discussed above do not follow clines along the continent or geographic regions, an observation that also supports the hypothesis of major founder and bottleneck effects and random genetic drift.

In order to test the hypothesis of HLA/KIR co-evolution we looked for correlations between the frequencies of KIR and their respective HLA ligands in the four Amerindian populations. For none of the three analyzed receptor-ligand pairs the observed frequencies differed from those expected under equilibrium (P>0.05). In addition, the observed frequencies of individual KIR and their known or putative HLA ligands were not correlated. These results are compatible with independent evolution and the hypothesis of neutral evolution of receptor-ligand combinations was not rejected. The combinations of KIR variants and specific variants of the cognate HLA ligands should be analyzed in future studies to refine the analysis in search of signatures of natural selection. Several examples of associations between *KIR*/*HLA* genotypes and variation of susceptibility to multifactorial diseases have been reported [37]–[41]. Some of these combined genotypes affect morbidity and mortality and thus fitness, therefore signs of natural selection could be revealed at the levels of both population and nucleotide/aminoacid sequence analyses, as reported for the *KIR3DL1/S1* polymorphism [42]. Negative correlation between the frequencies of activating KIR receptors and their HLA ligands was observed in an analysis of numerous populations worldwide and interpreted as evidence of natural selection [43]. Yet the fact that the difference between observed and expected frequencies of *KIR* genes and their HLA ligands in the present study was close to zero indicates that the selection pressure at least at this level is low. We reported a similar conclusion when analyzing a Brazilian urban population [11].

Internal nodes of a dendrogram based on genetic distances as performed in this study do not necessarily represent common ancestry. The similarities and differences between extant populations can be consequences of genetic drift, bottleneck, founder effect and other demographic factors and of natural selection, besides common ancestry. The limitations of the different typing methods also have to be considered, as they could be the reason of some unexpected grouping. Altogether, our results of both genetic distances (Figure 2) and principal component analyzes (Figure 3) are consistent with geography and common ancestry. However, although sharing a known common ancestry, the Guarani groups are more heterogeneous among themselves than different from other, less related, populations in both analyzes. This is another evidence of the effect of stochastic demographic factors during the evolutionary history of the Amerindian populations.

Although our data support the hypothesis of a major influence of demography on *KIR* gene frequencies in Amerindians, the absence of individuals who lack functional *KIR* recognition provides evidence that natural selection may also be involved in evolution of the *KIR* polymorphism in these populations. Our results are not suited to reject the hypothesis that natural selection may be acting on *KIR* evolution. However, based on our data, we conclude that demographic factors have been more important in shaping *KIR* gene frequencies in Amerindians than other evolutionary factors. Notwithstanding, analyzing isolated populations worldwide for both *KIR* and *HLA* diversity using different approaches would be a good step to bring more light to this discussion.

## Materials and Methods

### Ethics Statement

The coordinator of the project (MLPE) contacted the FUNAI (Fundação Nacional do Indio) and visited the indigenous areas to explain the purposes of the project and to obtain the consent from the authorities of the population. After permission, the research team went to the indigenous areas. Persons who were willing to participate came spontaneously to the health facility and were informed about the procedures and the purposes of the work. Participants provided verbal informed consent. Written consent was not required in 1988, when the samples were obtained. The ethics committee approved this procedure. The study was part of a protocol of population genetics of Amerindian groups approved by the Brazilian National Commission on Research Ethics (CONEP) under number 2046/2001.

### Study Populations

The study included 327 individuals from four different Amerindian populations from Brazil: Guarani M’byá (GRC, n = 81), Kaingang (KRC, n = 100), Guarani Kaiowá (GKW, n = 96) and Guarani Ñandeva (GND, n = 50). The GRC and KRC are from the indigenous area Rio das Cobras, municipality of Nova Laranjeiras, Paraná state (25°18′S, 52°32′W; 1,600 people, approximately two-thirds KRC and one-third GRC), living in different villages. The GKW are from the reservations areas of Amambai (23°06′S, 55°12′W; 4,500 people, among GKW and GND) and Limão Verde (23°12′S, 55°06′W; 460 people). GND are from Amambai and Porto Lindo (23°48′S, 54°30′W; 1,600 people) in southern Mato Grosso do Sul state, central Brazil. More information about these populations can be found in Petzl-Erler et al. (1993) [44] and Tsuneto et al. (2003) [45]. The study was part of a protocol of population genetics of Amerindian groups [45] approved by the Brazilian National Ethics Committee (CONEP) under number 2046/2001.

### *KIR* and *HLA* Typing

The typing of all *KIR* genes was performed by PCR-SSP with the primers sets that have been previously described [46], [47]. In case of inconsistencies between the two pairs of primers for the same gene we designed new primer pairs to validate the typing (Table 3). PCR was performed using 10 ng de DNA, 200 µM dNTP, 400 nM of each primer, 1.5 µM of MgCl_2_, 1× buffer and 0.25U of Taq Platinum DNA Polymerase (Invitrogen). Cycling conditions were: 2 min at 94°C; five cycles of 94°C for 5 sec, 65°C for 15 sec and 72°C for 30 sec; 21 cycles of 94°C for 5 sec, 60°C for 15 sec and 72°C for 30 sec; 4 cycles of 94°C for 5 sec, 55°C for 15 sec and 72°C for 30 sec. Gel electrophoresis on 3% agarose and ethidium bromide staining were used to visualize the presence or absence of the amplicons corresponding to each of the *KIR* genes and also to detect the group of non-functional alleles of *2DS4* characterized by a 22 pb deletion.

**Table 3:** Primers designed to solve discordant results obtained with previously described primer pairs.

Primer	sequence(5′→3′)	strand	position(nt)	size(bp)
KIR2DL1ex4mon	CCATCAGTCGCATGACG	coding	256	94^a^
KIR2DL1ex4jus	TCACTGGGAGCTGACAC	complementary		
KIR2DL2ex5mon	ACGGTTCTGGCAGGAGAGAG	coding	411	113^b^
KIR2DL2ex5jus	GGCCCTGCAGAGAACCTACA	complementary		
KIR2DS5ex4mon	ACGGTTCTGGCAGGAGAGAG	coding	183	87^c^
KIR2DS5ex4jus	GGCCCTGCAGAGAACCTACA	complementary		
KIR3DP1ex5mon	TCTCTCAGCCCAGCCGC	coding	676	104^e^
KIR3DP1ex5jus	CCCC**S**TCCCTTGATAGATGGTAG	complementary		
KIR3DL2ex4mon	CCAACTTCTCCATCGGTCCCT	coding	532	75^e^
KIR3DL2ex4jus	GGGAGTGAGGAACAGAACCATAA	complementary		
KIR3DL3ex4mon	GCAATGTTGGTCAGATGTCAG	coding	426	121^e^
KIR3DL3ex4jus	CATGGAATAGTTGACCTGGGAAC	complementary		
KIR3DL3ex3mon	CACTGTGGTGTCTGAAGGAC	coding	107	199^d^
KIR3DL3ex3jus	GGAGTGTGGGTGTGAACTG	complementary		

a modified from Gómez-Lozano & Vilches (2002) [54];

b modified from Uhrberg et al. (1997) [55];

c modified from Uhrberg et al. (2002) [16];

d modified from Du et al. (2007) [56];

e new.

The *HLA* typing was previously performed by PCR-SSOP ([48] and unpublished data).

### Statistical Analysis

The frequency of individuals carrying each *KIR* gene (PF = phenotypic frequency, or presence of the gene in either homozygosity or heterozygosity) and the phenotypic frequencies for the whole set of genes analyzed (*KIR* gene profile) were obtained by direct counting. Gene frequencies (GF) were estimated using Bernstein’s formula GF = 1−√(1−PF). The frequencies of haplogroups A and B were obtained by two approaches: (1) applying Bernstein’s formula; (2) after haplotype inference, by direct counting of haplotypes belonging to groups A and B.

Haplotype inference was performed using the software Haplo-IHP [36]. We inferred haplotypes using the *KIR* profile frequency data assuming that: (1) linkage disequilibrium patterns in these populations would be the same as previously described worldwide; (2) haplogroups or haplotypes widespread among populations, as haplogroup A, would exist also in the Amerindian populations analyzed. The haplotypes were assigned using the Haplo-IHP software that employs a greedy-EM hybrid algorithm. The expectation-maximization (EM) algorithm is an iterative procedure that uses unphased multilocus genotype frequencies along with the assumption of Hardy-Weinberg proportions to converge on final haplotype frequencies estimates. Additionally the Haplo-IHP software requires an *a priori* list of known/possible haplotypes as input. We used the list of haplotypes described previously [49] as the *a priori* haplotype list. As constraints for the estimation, we considered *3DL3* and *3DL2* present in all individuals and *2DL2*/*2DL3* and *3DL1*/*3DS1* as alleles of same locus. Haplotypes were also inferred manually, based on linkage disequilibrium and the segregation in some Amerindian families.

The *KIR* gene frequencies of the study populations and those of 57 worldwide distributed populations with distinct ancestries were used to calculate Ney’s [50] genetic distances. A neighbor-joining (NJ) dendrogram [51] was constructed based on these distances to represent the genetic relationship among populations. PHYLIP – Phylogeny Inference Package – version 3.6 [52] was used for these analyses and the dendrogram was visualized with the Treeview software [53]. Principal component analysis (PCA), a common technique used to summarize information from a large number of variables (gene frequencies) into a smaller set of variables (principal components), was also performed, using WinVista (http://forrest.psych.unc.edu/research/), to visualize populations genetic relationships by means of a two dimensional plot. The non-informative pseudogenes and framework genes were excluded from these analyses.

The expected frequency of co-occurrence of KIR and their HLA class I ligands was estimated and compared to the observed frequency using the chi-square test. The receptor/ligand pairs tested were KIR3DL1/S1+HLA Bw4, KIR 2DL2/3+HLA-C group 1, and KIR2DL1+HLA-C group 2. In addition, we counted in each individual the number of functional KIR–HLA combinations to estimate their average number and to determine the proportion of individuals in the population lacking functional receptor/ligand combinations.
