# Correction: *KIR* Gene Content in Amerindians Indicates Influence of Demographic Factors

**DOI:** 10.1371/journal.pone.0098427

**Published:** 2014-05-16

**Authors:** The PLOS ONE Staff

There are errors in Figure 1. The columns containing frequencies at the bottom of Figure 1A are in the wrong order. The authors have provided a corrected version here.

**Figure 1:**
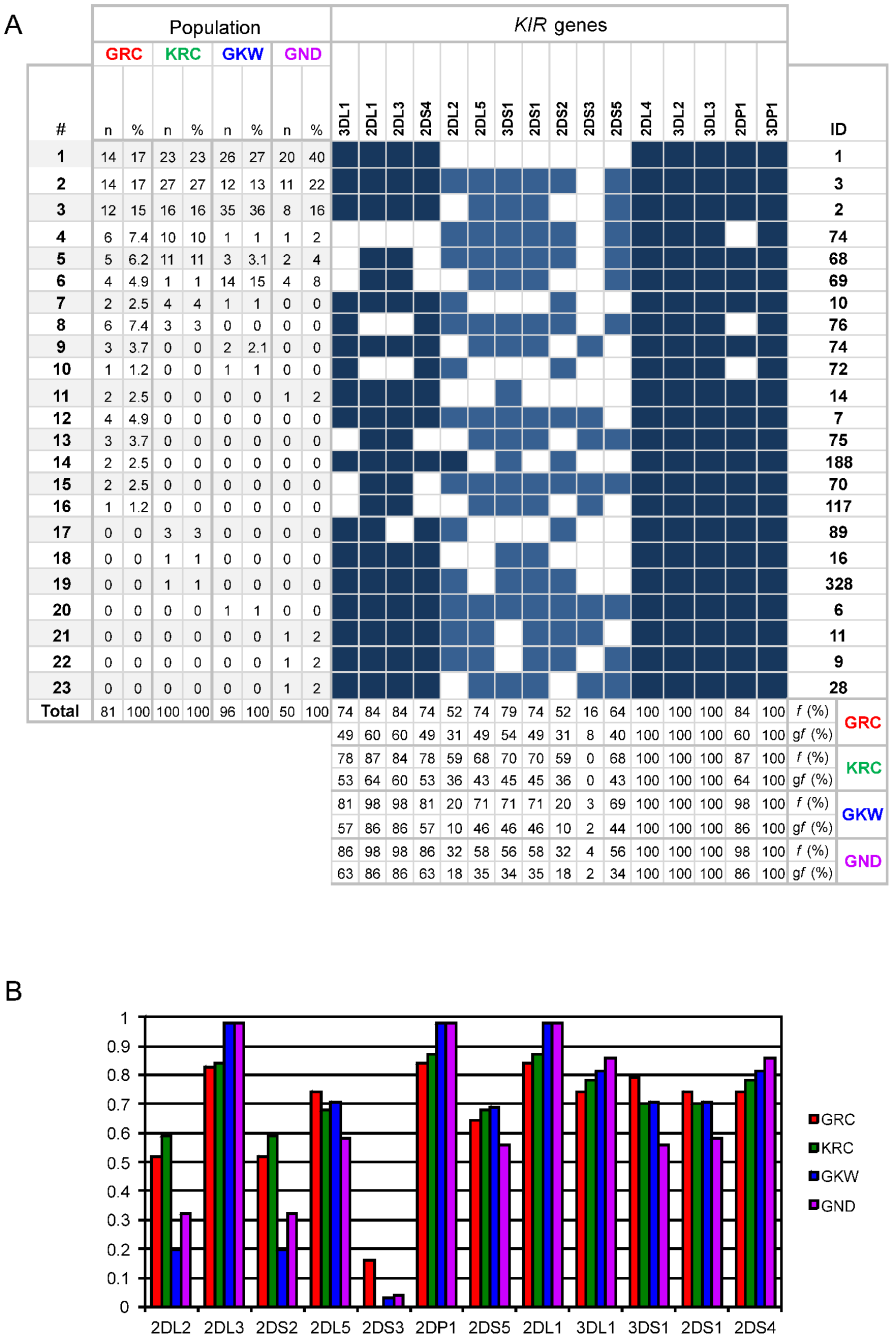
Frequency of  *KIR*  profiles and of individual  *KIR*  genes in the Kaingang and Guarani populations. **1A**  – The frequencies of KIR profiles are on the left side and the frequencies of individual genes are on the bottom. Filled boxes indicate presence of the gene and blank boxes, absence. In dark blue are genes typically from haplotypes A and light blue, from haplotypes B. n  =  number of individuals; *f*  =  carrier frequencies; *gf*  =  gene frequency; ID  =  identification number according allelefrequencies.net [24]. **1B** - Phenotypic frequencies of each KIR gene and pseudogene in the Kaingang and the three Guarani populations. Genes observed in all individuals are not shown.
